# Evaluation of several adjuvants in avian influenza vaccine to chickens and ducks

**DOI:** 10.1186/1743-422X-8-321

**Published:** 2011-06-26

**Authors:** Chun G Liu, Ming Liu, Fei Liu, Da F Liu, Yun Zhang, Wei Q Pan, Hao Chen, Chun H Wan, En C Sun, Hong T Li, Wen H Xiang

**Affiliations:** 1 Animal Influenza Laboratory of Agriculture Ministry, National Avian Influenza Reference Laboratory, OIE Avian Influenza Reference Laboratory, State Key Laboratory of Veterinary Biotechnology, Harbin Veterinary Research Institute of Chinese Academy of Agricultural Sciences, No.427 Maduan Street, Nangang District, Harbin, 150001, China; 2 College of Animal Science, Guizhou University, No. 207 Xiahui Road, Huaxi District, Guiyang, 550025, China; 3 State Key Laboratory of Veterinary Biotechnology, Harbin Veterinary Research Institute of Chinese Academy of Agricultural Sciences, No.427 Maduan Street, Nangang District, Harbin, 150001, China

## Abstract

The effects of three different adjuvants, mineral oil, Montanide™ ISA 70M VG, and Montanide™ ISA 206 VG, were evaluated on reverse genetics H5N3 avian influenza virus cell cultured vaccine. The immune results of SPF chickens after challenging with highly pathogenic avian influenza (HPAI) virus demonstrated that mineral oil adjuvant group and 70M adjuvant group provided 100% protection efficiency, but 206 adjuvant group provided only 40%. Statistical analysis indicated that the protection effects of mineral oil adjuvant group and the 70M adjuvant showed no significant difference to each other, but with significant difference to 206 adjuvant group. All three groups could induce high titres of antibody after immunizing SPF ducks, but there was no significant difference among them. The immunization effect of 70M adjuvant group on SPF chickens were the best and showed significant difference compared with optimized 70Mi Montanide™ eight series adjuvants groups. These results suggest that 70M adjuvant could be a novel adjuvant for preparing avian influenza vaccine.

## Introduction

H5 subtype highly pathogenic avian influenza (HPAI) viruses could cause severe disease and enormous economical loss to poultry farms. They could also cross the species barrier to infect mammals. In particular, the direct transmission of avian influenza viruses to humans without the pigs as "vessel" might be seriously endangered the poultry industry and human health [1-3]. It is very important to strengthen bird influenza surveillance, prevention and control work. The main control strategies for H5N1 subtype HPAI involve increased bio-security, surveillance, and vaccination, however, the vaccination is an effective and economic strategy in controlling the prevalence of this disastrous disease. At present, the widely used conventional inactivated H5 subtype bird influenza virus is from allantoic fluids of embryonated chicken eggs. It is necessary to solve many problems in vaccine development, including the difficulty in vaccine strains construction by conventional method [4], the serious byproducts pollution from embryonated chicken eggs in vaccines production progress, and the difficulty to differentiate nature infected and routine immunized birds. A new type of vaccine production system which could replace embryonated chicken eggs and differentiate between the infected and the immunized birds is urgent need.

The modern molecular biological techniques provide a new approach for new type of influenza vaccine design [5]. Compare to traditional vaccine development, the new influenza vaccine development trend should have the same or no less protective effects, saving times in the vaccine production process, alleviating environmental pollution, providing a higher levels of bio-security.

The reverse genetics H5N3 (rH5N3) avian influenza vaccine strain was successfully constructed by the reverse genetics technique [6]. The prepared rH5N3 vaccine strain, which could discriminate the infected birds from the immunized birds by N3 marker [7,8], can replicate effectively in MDCK cell lines [6]. To further promote vaccine effects and scan the optimal adjuvant, the rH5N3 virus was used in this study to evaluate different adjuvants.

## Materials and methods

### Virus

The rH5N3 avian influenza vaccine strain was previously constructed by reverse genetics [6]. Briefly, the six internal genes came from high-yield influenza virus A/Goose/Dalian/3/2001 (H9N2), hemagglutinin (HA) gene from A/Goose/HLJ/QFY/2004(H5N1), and neuraminidase (NA) gene from A/Duck/Germany/1215/73(H2N3) reference strain. The HA gene was modified by the deletion of four basic amino acids of the connecting peptide between HA1 and HA2. The rH5N3 was generated by co-transfection to mixed Madin-Darby canine kidney (MDCK) cell lines and Human embryonic kidney (HEK) 293T cell lines. Stock virus was made with MDCK cells. All experiments with H5N1 subtype influenza virus were performed in Bio-Safety Levels 3+ containment laboratory.

### Birds

The three-week-old specific pathogen free (SPF) White Leghorn chickens and SPF ducks were used in this experiment offered by Experimental Animal Center of the Harbin Veterinary Research Institute. All animals were housed in the stainless steel isolation cabinets that were ventilated under negative pressure with HEPA-filtered air.

### HI antigen and adjuvants

The H5 subtype avian influenza HI antigen and domestic mineral oil adjuvant provided by Harbin Wei Ke Biotechnology Development Company of the HVRI. Montanide™ ISA 206 VG adjuvant, Montanide™ ISA 70M VG adjuvant and Montanide™ ISA 70 essai Mi (i = 1-8) adjuvant provided by France Seppic Shanghai Branch [9].

### Preparation of vaccine

The cell-cultured rH5N3 avian influenza virus was inactivated by 1‰ formalin. The inactivated virus mixed with mineral oil adjuvant at 1:2 (Vol/Vol) and then emulsified as conventional methods. 70M, 70Mi, and 206 adjuvant vaccine prepared as described by Seppic protocols, respectively. Briefly, inactivated virus mixed with 70M and 70Mi adjuvant at a ratio of 2.6:7.4 (v/v), with 206 adjuvant at a ratio of 4.6:5.4 (v/v), respectively, and then emulsified as described by Seppic protocols.

### Immunization of animals

Immunization and challenge experiments were performed in accordance with instructions in the OIE manual to evaluate the effects of different adjuvant. The experiments were divided three parts. In the first part, all the fifty three-week-old SPF White Leghorn chickens were randomly divided into five groups equally. Three groups were immunized with mineral oil adjuvant vaccine, 70M adjuvant vaccine, and 206 vaccine adjuvant, respectively, the fourth group vaccinated only with inactivated virus without adjuvant, the fifth mock group injected with sterile phosphate-buffered saline (PBS). SPF chickens from group 1 to 4 vaccinated subcutaneously in the neck with 10 *μ*g/ml per chicken, and the mock group vaccinated with 0.3 ml PBS (Table 1).

**Table 1:** Experiment design

Parts	Birds	group	Boosting date ^*a*^	Bleeding date	Challenging date ^*b*^	Collection of swab ^*c*^
		Mineral oil				
		70M				
		70M				
Part I	SPF chickens	206		2, 3, 4, 6, 8, and 13 w after primary immunization	13 w after primary immunization	
		rH5N3 Antigen				
		PBS				
		70 M				
		206				
Part II	SPF ducks	rH5N3 Antigen	3 w p.v.^*d*^	0 day before initial immunization, 2, 6, and 9 w after primary immunization		
		PBS				
		70 M				
Part III	SPF chickens	70 Mi(i = 1-8)	3 w p.v.	2, 3, 4, 6, 8, and 13 w after primary immunization	13 w after primary immunization	3, 7, and 10 days p.c.^*e*^
		PBS				

^*a *^Group I didn't perform boosting immunization

^*b *^Group II didn't conduct challenge experiments.

^*c *^Group I didn't collected trachea and cloaca swabs after challenge.

^*d *^p.v.= post-vaccination

^*e *^p.c.= post-challenge

In part II experiments, thirty two three-week-old SPF ducks were randomly divided into four groups of eight birds each. Three groups were immunized with mineral oil adjuvant vaccine, 70M adjuvant vaccine, and 206 adjuvant vaccine with 0.25 ml antigen per duck in the neck, respectively. Group four as control injected with 0.5 ml PBS per duck. The boosting immunization was conducted three weeks after primary immunization with the same dose (Table 1).

In part III experiments, one hundred three-week-old SPF chickens were randomly divided into ten groups of ten birds. The first group was immunized with 70M adjuvant vaccine. The groups 2-9 inoculated with 70Mi (i = 1-8) adjuvant vaccine, respectively. SPF chickens from groups 2-9 vaccinated same as to the first group. Group 10 as control injected with 0.3 ml PBS each chicken. The boosting immunization was performed three weeks followed primary immunization with the same dose (Table 1).

### Monitoring of antibody titres

2, 3, 4, 6, 8, and 13 weeks after primary immunization, all birds were bled for sera collecting. 0 day before initial immunization, 2 weeks after primary immunization, 3 and 6 weeks after boosting immunization, all ducks were bled for sera collecting. The dynamic changes of antibody titres were detected by HI test.

### Challenge

Each chicken from five groups of part I and ten groups of part III were challenged with H5N1 HPAI virus A/Gs/HLJ/QFY/04(H5N1) 10^6 ^EID_50 _through intranasal and eye drop administration. Adjuvant effects for rH5N3 vaccines were evaluated after 13 weeks post-vaccination. Morbidity and mortality was observed daily for 10 days. Cloaca and throat swabs of birds of part III were collected at 3, 7, and 10 days post-challenge for virus detection.

### Statistical analysis

A Student t-test was used to analyze the results of the HI assay for the levels of antibodies.

## Results

### Dynamic changes of antibody titres

**Figure 1:**
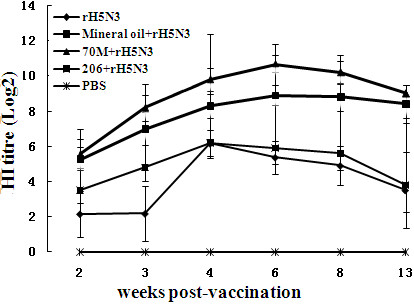
**Antibody responses to mineral oil-adjuvanted, 70M-adjuvanted, 206-adjuvanted or non-adjuvanted rH5N3 vaccines in SPF chickens**.

The antibody titres of five groups chickens at 2, 3, 4, 6, 8, and 13 weeks post-vaccination in part I were measured by HI test (Figure 1). There was no significant difference (*P *> 0.05) between mineral oil adjuvant group and 70M adjuvant group from statistical analysis, but there were significant differences (*P *< 0.01) when mineral oil adjuvant group or 70M adjuvant group compared with 206 adjuvant group. The antibody titre from 70M adjuvant group was higher than that of the mineral oil adjuvant group. The chicken of control groups did not elicit HI antibody.

**Figure 2:**
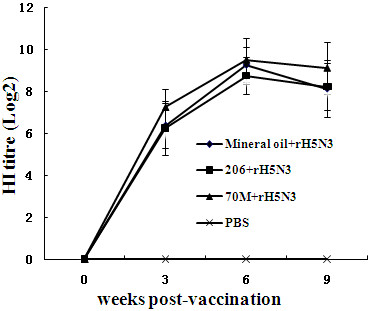
**Antibody responses to mineral oil-adjuvanted, 70M-adjuvanted, 206-adjuvanted, and non-adjuvanted rH5N3 vaccines in SPF ducks**.

0 day before initial immunization, 3 weeks after primary immunization, 3 and 6 weeks post boosting immunization, the ducks antibody titres of four groups in part II were detected by HI test (Figure 2). The results indicated that all three groups can induce good immune response and produce similar level of HI antibody. There were no statistically significant differences (*P *> 0.05) among mineral oil adjuvant group, 70M adjuvant group, and 206 adjuvant group. The ducks of control groups did not elicit HI antibody.

**Figure 3:**
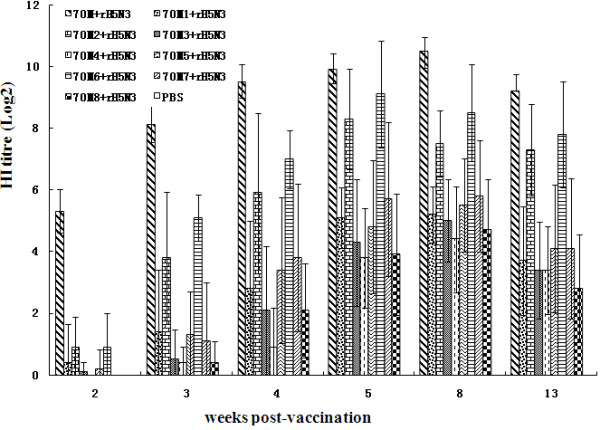
**Antibody responses to 70M-ajuvanted or 70Mi-adjuvanted rH5N3 vaccines in SPF chickens**.

The antibody titres of 2, 3, 4, 6, 8, and 13 weeks post-immunization in part III chickens immunized with 70M or 70Mi (i = 1-8) adjuvant vaccine were monitored by HI test (Figure 3). There are statistically significant differences (*P *< 0.01) between 70M adjuvant group and other 70Mi (i = 1-8) groups, but each of nine groups showed no statistically distinguishable differences (*P *> 0.05) compared with 206 adjuvant group. The chicken of control groups did not elicit HI antibody.

Birds had inflammation at injection site in mineral oil adjuvant group, but other groups did not show any clinical and pathological signs.

### Study of challenge and protection

After challenge with A/Gs/HLJ/QFY/04 (H5N1) virulent viruses, chickens from mineral oil adjuvant and 70 M adjuvant vaccine groups in part I showed no sign of disease and no birds died in this experiment. Mineral oil adjuvant vaccine or 70 M adjuvant vaccine were able to provide 100% protective efficiency to SPF chickens (Table 2). However, 206 adjuvant vaccine group and antigen without adjuvant group provide only 50% or even less protective efficiency to SPF chickens. Chickens from the control group were dead in two days after challenge.

**Table 2:** The HI antibody titres of chickens of Part I post-vaccination and protection efficiency post-challenge

groups	The HI antibody titre(log2) p.v. ^*a*^						Sick/Dead/Total	The HI antibody titre(log2) p.c. ^*b*^
			
	2 w	3 w	4 w	6 w	8 w	13 w		
Mineral oil	5.3	7.0	8.3	8.9	8.8	8.4	0/0/10	8.8
70 M	5.6	8.2	9.8	10.7	10.2	9.0	0/0/10	9.0
206	3.5	4.8	6.2	5.9	5.6	3.8	4/6/10	8.9
rH5N3 Antigen	2.2	2.2	6.2	5.4	4.9	3.5	6/5/10	9.7
PBS	0	0	0	0	0	0	10/10/10	-- ^*c*^

^*a *^Presented as log2 HI titres. Antibody titres were determined by HI test in which the test serum had been diluted twofold,

^*b *^Presented as log2 HI titres. Antibody titres on 10 day p.i. were determined by HI test in which the test serum had been diluted twofold.

^*c *^Birds were died in 2 days p.c.

In the part III, the three groups of 70M adjuvant vaccine, 70M2 adjuvant vaccine and 70M6 adjuvant vaccines provided 100% protect to chickens with no virus shedding, no sick sign, no dead post-challenge. However, all other vaccine groups cannot provide complete protection to SPF chickens and also there were virus shedding, sick sign, and even death of birds among these groups. The chickens from mock group were all dead within two days after challenge (Table 3).

**Table 3:** The HI antibody titre of chickens in Part III post-vaccination and protection efficiency post-challenge

Groups	The antibody HI titre (log2) p.v. ^*a*^						Virus shedding/Dead/Total	The HI antibody titre(log2) p.c. ^*b*^
			
	2 w	3 w	4 w	5 w	8 w	13 w		
70 M	5.3	8.1	9.5	9.9	10.5	9.2	0/0/10	9.1
70M1	0.4	1.4	2.8	5.1	5.2	3.7	5/2/10	9.4
70M2	0.9	3.8	5.9	8.3	7.5	7.3	0/0/10	8.8
70M3	0.1	0.5	2.1	4.3	5.0	3.4	5/1/10	8.4
70M4	0	0.4	0.9	3.8	4.4	3.4	6/1/10	5.9
70M5	0.2	1.3	3.4	4.8	5.5	4.1	2/1/10	6.8
70M6	0.9	5.1	7.0	9.1	8.5	7.8	0/0/10	8.1
70M7	0	1.1	3.8	5.7	5.8	4.1	6/2/10	9.3
70M8	0	0.4	2.1	3.9	4.7	2.8	2/2/10	7.9
PBS	0	0	0	0	0	0	10/10/10	-- ^*c*^

^*a, b *^and ^*c *^are same to table 2.

## Discussion

An effective vaccine needs not only good antigens but also preferable adjuvant to enhance the immunogenicity of antigen. The adjuvant was used to enhance humoral and cellular immune responses, but adjuvant would also lead to side-effect to bodies, such as inflammation, tissue damage and pain [10]. Oil emulsion vaccine could promote antibody titre and extend immunity period. However, mineral oil long term stand at the injection site, and caused inflammation and local tissue necrosis, lead commercial value of the birds lower.

In present experiment, mineral oil, 70M, and 206 adjuvant were used to evaluate adjuvant effects. 70M adjuvant and 206 adjuvant were oil in water (W/O) and water in oil (W/O/W) adjuvant based on purified mineral, respectively. The previous studies showed that the vaccine combined 70M adjuvant, Freund's complete adjuvant, or incomplete Freund's adjuvant were able to induce similar antibody responses, but 70M adjuvant vaccine caused obviously much less inflammatory response after inoculation [11-14]. The avian influenza vaccine emulsified with 70M adjuvant prepared provided a good protection to immunized chicken [15-17]. Vaccine conjugated 70M adjuvant induced not only humoral immune responses, but also strong cellular immune responses after immunizing mice [18]. 70M and 206 adjuvant could provide the immune enhancing effects on Eimeria acervulina vaccine [19].

In this experiment, the antibodies of SPF chicken induced by 70M adjuvant vaccine or mineral oil adjuvant vaccine were higher than that of protective threshold two weeks post-inoculation, but 206 adjuvant vaccine and antigen without adjuvant did not. The antibody levels elicited by 206 adjuvant group achieved protective threshold three weeks later and reached the highest level after four weeks then began to decline, but it was no longer able to provide theoretical protection 13 weeks after immunization, which is consist with porcine circle virus disease inactive vaccine [20]. In part I and II experiments, the antibody titres of 70M adjuvant group were higher than that of the mineral oil adjuvant group, but the differences between two groups were not significant (P > 0.05) statistically, while the two groups showed significant differences (P < 0.01) compared to 206 adjuvant group in part I but not in part II experiments. In part II, 206 adjuvant group induced the similar immune responses to 70M adjuvant group and mineral oil adjuvant group of ducks.

We speculated that 206 adjuvant was a kind O/W type adjuvant, it released into the lymphoid tissue rapidly after immunization and leaded to inefficiency to chickens, while 70M adjuvant and mineral oil adjuvant were a type of W/O adjuvant and could be stored at the injection site and release slowly. Another reason maybe due to the chickens, ducks and pigs are different species, their immune systems possessed differences leading to the distinguishing immune responses elicited by 206 adjuvant vaccines. However, the real reason was unknown and to be further studied.

Since 70 M adjuvant induced better immune effects to the chickens and ducks, the French Seppic company optimized 70M adjuvant and prepared eight 70M Series of adjuvant, Montanide™ ISA 70 essai Mi (i = 1-8) adjuvants. Immunity and challenge experiments to SPF chickens showed that the antibody induced by 70M adjuvant group surpassed protective threshold two weeks after immunization, while the other groups were lower than that. Three weeks later, only 70M6 adjuvant group achieved that level. All the other vaccine groups (70 M1, 70M3-5, 70M7, and 70M8), the titres of antibody stay low from two weeks to thirteen weeks post-vaccination, and to 13 weeks each groups antibody titre was lower than the threshold while 70M2 and 70M6 adjuvant groups antibody levels remained at 7log2 above, 70M adjuvant group reached to 9log2 above. 70M2 and 70M6 adjuvant groups achieved to the highest levels of antibody at six weeks post-immunization, and the other groups were at eight weeks post-immunization. Challenge experiment results showed that 70M, 70M2, and 70M6 adjuvant groups could provide 100% protection. The results suggested that 70M adjuvant and 70M6 adjuvant groups had good effects in all groups, and 70M adjuvant group was the best one with statistically significant differences (P < 0.01) to all other groups.

There were no visible clinical and pathological signs at birds inoculation sites two weeks after immunization of the 70M adjuvant vaccine, 70Mi Series adjuvant vaccines, and 206 adjuvant vaccines, suggesting that all these Seppic adjuvant had smaller side reactions. 70M adjuvant could be best as a new type of adjuvant applied in the production of avian influenza vaccines.

## Competing interests

The authors declare that they have no competing interests.

## Authors' contributions

CGL organized the whole process, took part in all the experiments and drafted the manuscript. ML and YZ designed the whole project and edited the manuscript. FL and DFL performed the Statistical analysis. CHW and ECS carried out the animal experiment. WQP and HC participated in detection of HI antibody. WHX participated in the design and coordination of this study. All authors read and approved the final manuscript.
